# Preparation and Photocatalytic Properties of Anatase TiO_2_ with Hollow Hexagonal Frame Structure

**DOI:** 10.3390/nano12091409

**Published:** 2022-04-20

**Authors:** Mengyuan Teng, Haixia Liu, Bensheng Lin, Xiangzhu Zhou, Wei Zhou

**Affiliations:** Shandong Provincial Key Laboratory of Molecular Engineering, School of Chemistry and Chemical Engineering, Qilu University of Technology (Shandong Academy of Sciences), Jinan 250353, China; tengmy97701@163.com (M.T.); linbensheng2022@163.com (B.L.); zhouxiangzhu521@163.com (X.Z.)

**Keywords:** HTiOF_3_, anatase TiO_2_, hexagonal frame structure, photocatalytic, photocatalytic hydrogen evolution

## Abstract

Titanium dioxide (TiO_2_) has been widely used to solve energy and environmental pollution problems due to its excellent properties. In this study, the precursor (HTiOF_3_) with a spherical structure composed of hexagonal prisms was prepared via a simple solvothermal method using tetrabutyl titanate, hydrofluoric acid, glacial acetic acid and isopropanol as raw materials. Then, the calcination time and temperature of the precursor were controlled to prepare anatase TiO_2_ with different morphologies, and the photocatalytic performance of the prepared catalysts was studied. When the precursor was calcined at 600 °C for 7 h, the prepared TiO_2_ had a unique hexagonal framework structure and exhibited excellent photocatalytic performance. The degradation rate of the RhB solution was 98.58% at 40 min and the rate of hydrogen evolution was 2508.12 μmol g^−1^ h^−1^.

## 1. Introduction

TiO_2_ as an essential component of inorganic semiconductor catalysts has been attracting attention for a long time. It is considered to be an effective, environmentally benign and simple synthetic semiconductor photocatalytic material. It has been widely applied to many fields, such as photocatalysis, environmental pollution control and new energy conversion [1,2,3,4,5,6]. For most pragmatic applications of TiO_2_, it is of great importance to have exact control of the morphology, size, crystal phase and specific surface area because the properties of TiO_2_ strongly depend on these structural characteristics [7,8,9,10,11,12,13]. In particular, there are more and more researchers focusing their attention on the controllable synthesis of anatase TiO_2_, which shows a higher photoactivity than brookite or rutile [14].

In recent years, multifarious anatase TiO_2_ with different morphologies were reported, such as nanorods, nanoflowers and nanotubes [15,16,17]. Many researchers have put in a great deal of effort to explore the synthesis of various TiO_2_ nanostructures. However, most synthetic strategies have relied on the complex processes of experiments or the use of templating agents. For example, Klara Hernadi’s group [18] reported the synthesis of TiO_2_ hollow spheres via a hydrothermal and annealing route. However, there are carbon spheres as an aid to the template in this process. Lu et al. [19] prepared nanoporous TiO_2_ via a complicated route, which required two steps to prepare. Therefore, researchers are working on a simple and effective method to synthesize TiO_2_ nanostructures with unique morphology to improve the photocatalytic performance of the samples [20,21].

More and more effort has been devoted to developing various hollow titanium dioxide materials with different sizes, shapes and polymorphs due to the advantages of hollow nanostructures. However, these materials are all based on spherical structures. There have rarely been reports about single-crystal TiO_2_ materials with a hexagonal morphology. In addition, most of the hollow structures are completed through HF engraving or a basic framework of polymeric materials [22,23]. It is a big challenge for researchers to synthesize non-spherical hollow frame nanomaterials.

In this work, anatase TiO_2_ with a hollow hexagonal frame structure was synthesized using a template-free method. First, the precursor (HTiOF_3_) with a spherical structure composed of hexagonal prisms was synthesized using the hydrothermal method. The effect of changing the type of solvent on the precursor morphology was investigated. Then, the precursor was converted to TiO_2_ via calcination. TiO_2_ with different morphologies could be obtained by changing the calcination temperature or time, and the photocatalytic performance could be affected by the different morphologies. The results showed that the hollow hexagonal frame TiO_2_ had excellent photocatalytic activity and good stability.

## 2. Materials and Methods

### 2.1. Materials

The starting materials that were utilized were tetrabutyl titanate (TBT, chemical purity grade, Shanghai Macklin Biochemical Co., Ltd., Shanghai, China), titanium tetraisopropanolate (Shanghai Macklin Biochemical Co., Ltd., Shanghai, China), acetic acid and isopropanol (Tianjin Fuyu Fine Chemical Co., Ltd., Tianjin, China) and hydrofluoric (Shanghai Macklin Biochemical Co., Ltd., Shanghai, China). All chemicals were used as received without further purification. Distilled water was used throughout the experiment.

### 2.2. Synthetic Process

First, acetic acid (28 mL, 9.2 M) and isopropanol (21 mL, 4.9 M) were measured and mixed well under magnetic stirring. Then, hydrofluoric acid (1.6 mL, 0.7 M) was added to the mixed solution at room temperature. Tetrabutyl titanate (0.01 mol) was added dropwise to the above-mixed solution and stirred for 30 min to blend well. The stirred solution was transferred to a Teflon-lined stainless steel autoclave (100 mL) and reacted at 180 °C for 10 h. In order to remove unreacted precursor in the solution, the product was rinsed 3 times with deionized water (20 mL) and then rinsed with ethanol (20 mL) 3 times. The product was collected via centrifugation (5 min per cycle at 8000 rpm). Finally, the product was dried at 60 °C overnight. The dried precursor was calcined at 600 °C for 7 h (600 °C/7 h) with a heating rate of 2 °C/min to obtain hollow hexagonal frame TiO_2_. The precursor was calcined with the same heating rate for 2 h at 100 °C (100 °C/2 h), 300 °C (300 °C/2 h) and 600 °C (600 °C/2 h) to explore the effect of calcination temperature and time on the morphology and properties. The effect on the final morphology of the precursor was explored by changing a variable in the reaction conditions while other reaction conditions were unchanged (Table 1).

### 2.3. Characterization

X-ray diffraction (XRD) data were tested using a Bruker D8 Advanced X-ray powder diffractometer (Rigaku Corp, Tokyo, Japan) with Cu-Ka radiation (λ = 1.5418 Å). The morphologies of the as-prepared samples were analyzed using scanning electron microscopy (SEM, S-4800, Hitachi, Tokyo, Japan). The UV-vis diffuse reflectance spectra (DRS) were acquired on a UV-2550 spectrophotometer (Shimadzu, Tokyo, Japan). Photoluminescence (PL) spectra were measured at room temperature using a spectrophotometer (F97Pro, Lengguang, Shanghai, China). The N_2_ adsorption and desorption tests of samples were undertaken on an Autorsorb-iQ instrument (Qutachrome, Boynton Beach, FL, USA). The specific surface area was calculated using the Brunauer–Emmett–Teller (BET) method. Thermogravimetric analysis (TG) was performed in a nitrogen (N_2_) atmosphere using a thermogravimetric analyzer (Q50, Waters, Milford, MA, USA) at a heating rate of 5 °C min^−1^.

### 2.4. Photoelectrochemical Test

The transient photocurrent response and electrochemical impedance were measured using 0.5 M Na_2_SO_4_ aqueous electrolyte on a CHI 760E (Shanghai, China) electrochemical workstation in a standard three-electrode system. Then, 10 mg catalysts were dissolved in a mixed solution of 0.98 mL ethanol and 0.02 mL Nafion (5.0 wt%) and then given an ultrasonic treatment for 1 h. Next, a 5 μL mixed solution was loaded on a glassy carbon electrode and dried to obtain a working electrode. Pt was used as the counter electrode and Ag/AgCl as the reference electrode. A 300 W Xe lamp (PLS-SXE300 UV, Beijing Perfectlight Co., Ltd., Being, China) was utilized as the light source.

### 2.5. Measurement of Photocatalytic Activity

In this process, Rhodamine B (RhB) dye was used to evaluate the photocatalytic activity of all samples [24]. First, 0.1 g synthetic catalyst was added to 100 mL RhB (20 mg/L) aqueous solution. In order to obtain an equilibrium adsorption state, the mixture was magnetically stirred for 30 min in the dark before irradiation. A 300 W xenon lamp (PLS-SXE300UV, Beijing Perfectlight Co., Ltd., Beijing, China) was used as the light source. Then, 3 mL reaction solution was taken out every 10 min under simulated sunlight and the catalyst was filtered out. Finally, the RhB solution was determined using UV-vis spectroscopy. Under the same experimental conditions, 0.05 g, 0.15 g and 0.2 g of the catalyst were added to 100 mL of RhB solution for comparative experiments to study the effect of catalyst addition on the degradation efficiency.

### 2.6. Measurement of Photocatalytic H_2_ Evolution

The photocatalytic H_2_ evolution test was performed according to the method reported by Xia et al. [25]. First, 50 mg catalyst was dispersed in 40 mL water and 10 mL methanol. Then, 1 wt% of Pt provided by H_2_PtCl_6_·6H_2_O was added to the mixed solution as a co-catalyst. The reaction device was sealed and N_2_ was used to purge the solution for 30 min. Under the ultraviolet light source provided by a 300 W Xe lamp (PLS-SXE300 UV, Beijing Perfectlight Co., Ltd., Beijing, China), hydrogen was generated via irradiation. The entire reaction was kept at 15 °C. A gas chromatograph with N_2_ as the carrier gas was used to detect the hourly H_2_ production.

## 3. Results

**Figure 1:**
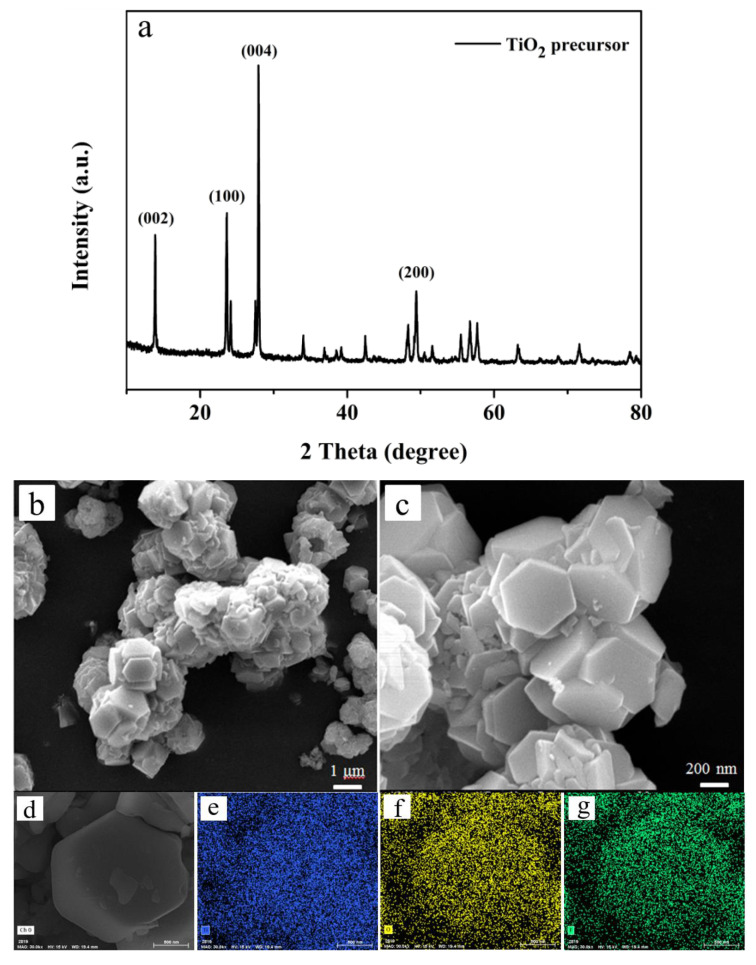
(**a**) XRD patterns; (**b**,**c**) SEM micrographs of TiO_2_ precursor; (**d**–**g**) EDS images of TiO_2_ precursor: Ti (blue), O (yellow) and F (green).

The TiO_2_ precursor (S1) obtained through the solvothermal reaction at 180 °C for 10 h was characterized using XRD and SEM. The corresponding XRD patterns are displayed in Figure 1a. Unexpectedly, the peaks of as-prepared samples in the XRD patterns were not in accordance with the anatase TiO_2_ structure (JCPDS NO.21-1272). It can be seen from the figure that the sample had diffraction peaks at 2θ = 13.92°, 23.67°, 28.10° and 48.40°, which corresponded to the {002}, {100}, {004} and {200} planes of HTiOF_3_ [26], respectively, and it could be preliminarily determined that the TiO_2_ precursor was HTiOF_3_. According to the energy-dispersive spectrometer (EDS) spectrum (Figure 1d,g), the sample was identified as a titanate compound, which included Ti, F and O elements. It could be further verified that the TiO_2_ precursor was HTiOF_3_. Subsequently, the as-prepared hexagonal TiO_2_ precursor nanospheres were characterized via scanning electron microscopy. It can be seen in Figure 1b,c that the precursor exhibited a spherical structure assembled from many hexagonal prisms with smooth surfaces. The size of each hexagonal prism was approximately 2–3 μm.

**Figure 2:**
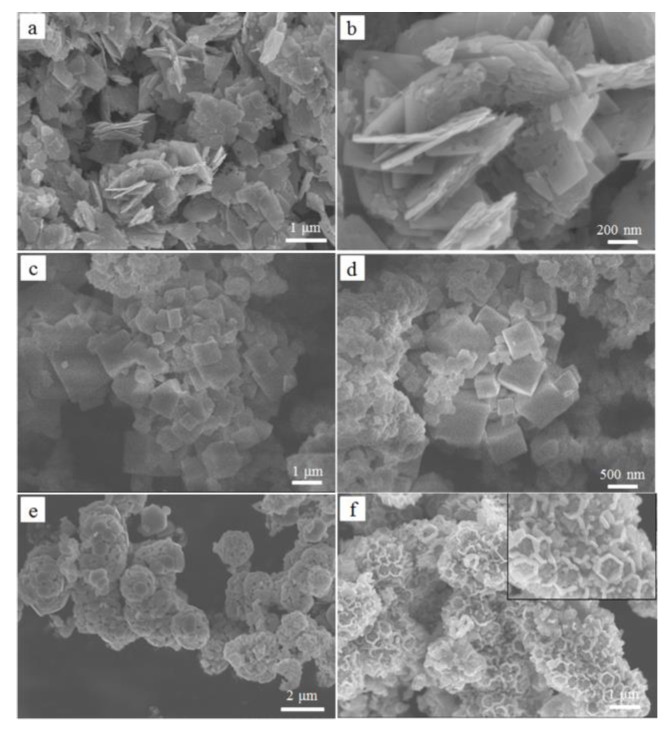
SEM image of samples prepared under different reaction conditions: (**a**,**b**) S2; (**c**,**d**) S3; (**e**,**f**) S5.

In order to explore the effects of different reaction conditions on the morphology of the samples with hexagonal structures, a series of experiments were also carried out by adjusting different solvents (Table 1). The crucial role of HAc is to inhibit the hydrolysis and morphological control agents of TBT. HAc played a vital role in determining the morphology of the hexagonal structure of HTiOF_3_ [27]. This was well demonstrated in the S2 experiment. When only HAc was not added, sample S2 exhibited sheet-like structures, as shown in Figure 2a,b. Furthermore, when isopropanol was not used, only some bulky and irregularly shaped block structures were found, as shown in Figure 2c,d. When HF was not added, no significant morphology was observed in the prepared sample (this sample is not shown here). This indicated that the special morphology of the hexagonal TiO_2_ structure could not be formed in the case of lacking one of the reactants. In addition, when the titanium source was changed from tetrabutyl titanate to titanium isopropoxide, the sample’s morphology was still hexagonal microsphere structures (Figure 2e,f). It demonstrated that the type of titanium source did not affect the morphology of the as-prepared sample.

**Figure 3:**
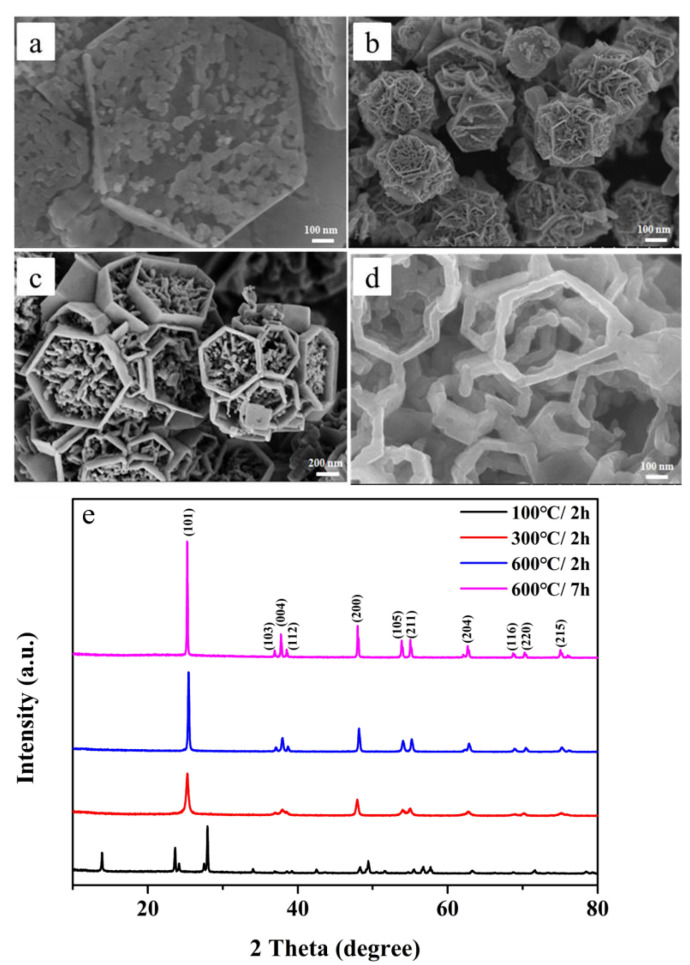
SEM images of TiO_2_ synthesized with different calcined temperatures: (**a**) 100 °C/2 h; (**b**) 300 °C/2 h; (**c**) 600 °C/2 h; (**d**) 600 °C/7 h. (**e**) XRD patterns of TiO_2_ synthesized with different calcined conditions. Note: 100 °C/2 h, the precursor calcined at 100 °C for 2 h; 300 °C/2 h, the precursor calcined at 300 °C for 2 h; 600 °C/2 h, the precursor calcined 600 °C for 2 h; 600 °C/7 h, the precursor calcined 600 °C for 7 h.

Furthermore, in order to obtain TiO_2_, the as-prepared precursor (HTiOF_3_) was calcined at different temperatures. As illustrated in Figure 3a, when the precursor was calcinated at 100 °C for 2 h, the hexagonal smooth surfaces gradually disappeared. The reason for this phenomenon was that HTiOF_3_ decomposed during the calcination process to generate HF gas and the overflow of the gas caused the surface to be uneven [26,28]. When the calcination temperature was raised to 300 °C, the end face of the hexagonal prism disappeared and six edges became apparent. All the side faces of the hexagonal prism were not broken and kept their original state (as shown in Figure 3b). Figure 3c is the SEM image of the precursor after calcination at 600 °C for 2 h. The samples gradually grew into hexagonal box structures. However, it was found that some material still existed in hexagonal boxes. The hexagonal frame structure was not obtained until the annealing reached 600 °C for 7 h. As shown in Figure 3d, the compounds in the hexagonal boxes wholly disappeared and the frame structure of TiO_2_ could be seen. The side face of the sample still maintained the previous complete appearance.

The corresponding XRD patterns for each sample are shown in Figure 3e. When the calcination temperature and the calcination time were 100 °C and 2 h, respectively, the precursor was not completely converted into TiO_2_ due to insufficient calcination. Therefore, it could be seen that the diffraction peak of the 100 °C/2 h sample corresponded to HTiOF_3_. No impurity peaks of other phases were found in the diffraction peaks of the other three samples. The characteristic peaks at 2θ values of 25.28°, 36.95°, 37.8°, 38.58°, 48.05°, 53.89°, 55.96°, 62.69°, 68.76°, 70.31° and 75.03° could be assigned to the (101), (103), (004), (112), (200), (105), (211), (204), (116), (220) and (215) crystal planes of anatase TiO_2_ (JCPDS No. 21-1272) [29]. In addition, with the increase in calcination temperature and calcination time, the diffraction peaks of the samples became sharper and sharper, which indicated that the crystallization degree was getting higher. Thus, it could be concluded that the formation of the hexagonal frame structure was not only related to the calcination temperature but also to the calcination time.

**Figure 4:**
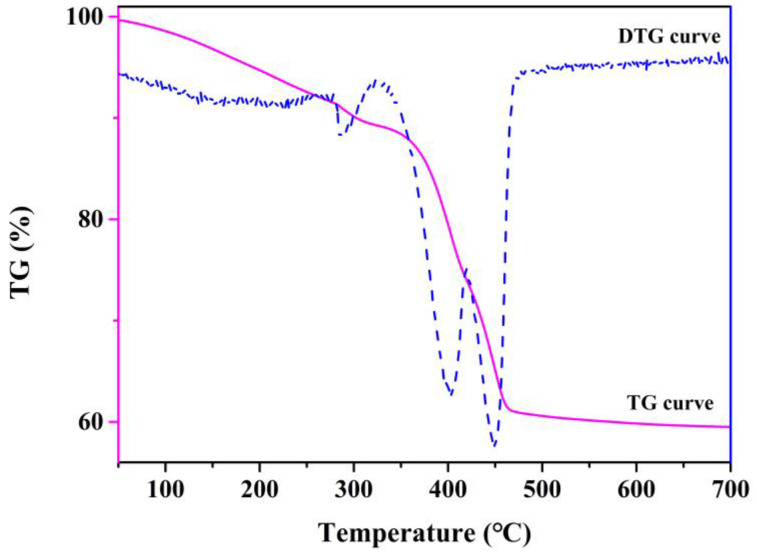
The TG and DTG curves of the TiO_2_ precursor (HTiOF_3_).

The TG and DTG curves of precursor (HTiOF_3_) are shown in Figure 4. It can be seen that there were four processes in the transformation from the HTiOF_3_ to TiO_2_. The first stage below 280 °C was a slight weight loss, which was mainly caused by the deintercalation of physically adsorbed water on the surface of the sample and the disappearance of evaporation of other chemicals. In the second stage of 280–400 °C, HTiOF_3_ was decomposed and converted into TiOF_2_, and some of the generated HF escaped. The third stage was 400–450 °C, where the decomposition of some titanium-containing impurities continued [30]. The fourth stage was 450–700 °C, in which TiOF_2_ was completely converted into TiO_2_. The result was consistent with the description of the SEM in Figure 3a,d.

**Figure 5:**
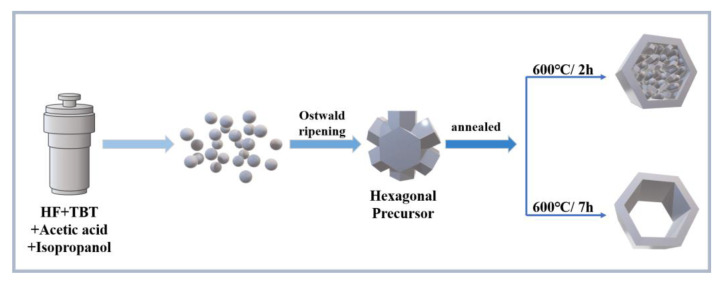
The schematic diagram of TiO_2_ with a hexagonal frame structure. Note: 600 °C/2 h, the precursor calcined 600 °C for 2 h; 600 °C/7 h, the precursor calcined 600 °C for 7 h.

Based on the above experimental exploration and characterization analysis, the formation mechanism of the obtained TiO_2_ with hexagonal frame structure was discussed. This process could basically be divided into the following stages: (I) hydrolysis of tetrabutyl titanate, (II) formation of spherical morphology composed of hexagonal structures, (III) Ostwald ripening and (IV) formation of TiO_2_ with hexagonal frame structures. First, the hydrolysis reaction of tetrabutyl titanate occurred and the nanoparticles mainly composed of the titanate compound were formed under solvothermal conditions. Subsequently, the nanoparticles aggregated into spherical structures assembled from hexagonal prisms. Next, the hexagonal prisms underwent Ostwald ripening and recrystallization growth due to annealing at a high temperature. Finally, the end face disappeared and the TiO_2_ with a hexagonal frame structure was obtained. It is worth noting that the side faces of the hexagonal prism were not broken and retained the complete morphology. The specific formation process of the hexagonal frame structure TiO_2_ is shown in Figure 5. The reaction equations for this process were as follows:

Ti(OR)_4_ + 4R-OH→Ti(OH)_4_ + 4R-OR (1)

Ti(OH)_4_ + 3HF→HTiOF_3_ + 3H_2_O (2)

2HTiOF_3_→TiO_2_ + 2HF + TiF_4_ (3)

**Figure 6:**
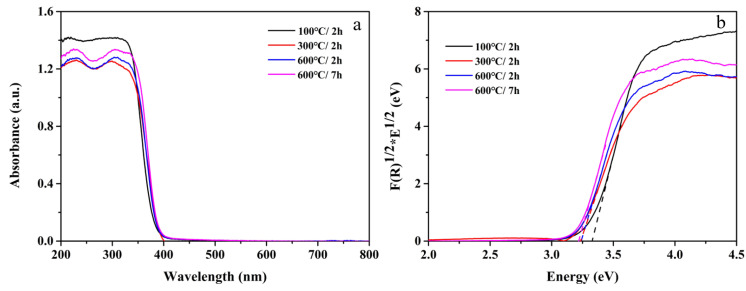
(**a**) UV-vis absorption spectra of four samples and (**b**) tauc plot. Note: 100 °C/2 h, the precursor calcined at 100 °C for 2 h; 300 °C/2 h, the precursor calcined at 300 °C for 2 h; 600 °C/2 h, the precursor calcined 600 °C for 2 h; 600 °C/7 h, the precursor calcined 600 °C for 7 h.

For the purpose of studying the light absorption properties of four samples, the UV-vis diffuse reflectance spectra of four samples were investigated separately. It could be seen that all samples had no absorption in the visible light region, which was mainly caused by the large band gap [31]. With the increase in calcination temperature and calcination time, the light absorption intensity of the 600 °C/7 h sample was the highest. Compared with anatase TiO_2_, HTiOF_3_ had a higher UV absorption intensity. In addition, Figure 6b shows a tauc plot, which was obtained using a Kubelka–Munk transformation to determine the band gaps of TiO_2_ and HTiOF_3_ [32]. The band gap of 600 °C/7 h was 3.22 eV, which was smaller than other samples. It was shown that the difference in the morphology of TiO_2_ also caused a slight change in the band gap. The band gap of 100 °C/2 h was 3.3 eV. Although the UV absorption intensity of TiO_2_ was not as high as that of HTiOF_3_, the narrow band gap of TiO_2_ showed that it had better utilization of UV-vis light [33].

**Figure 7:**
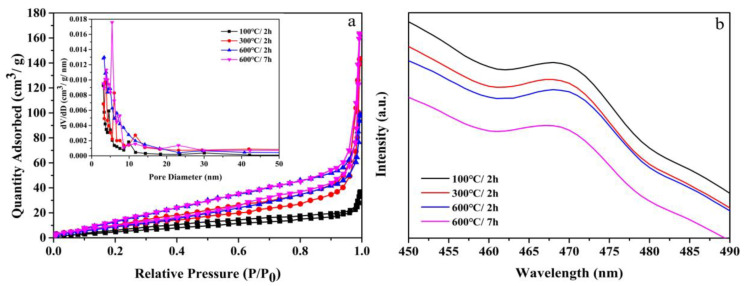
(**a**) N_2_ adsorption and desorption isotherms and pore size distribution curves (inset) of all samples and (**b**) PL spectra for four samples at room temperature. Note: 100 °C/2 h, the precursor calcined at 100 °C for 2 h; 300 °C/2 h, the precursor calcined at 300 °C for 2 h; 600 °C/2 h, the precursor calcined 600 °C for 2 h; 600 °C/7 h, the precursor calcined 600 °C for 7 h.

Samples with different morphologies have different specific surface areas and pore structures, which will affect photocatalytic performance. The N_2_ adsorption and desorption isotherms and pore size distribution curves are shown in Figure 7a. The isotherms of the four samples were all type IV isotherms, indicating a mesoporous structure [34]. It was shown that the samples had a mesoporous structure and pore diameters between 2 and 50 nm. The calculated values of the BET surface area and pore volume are listed in Table 2. It can be seen that 600 °C/7 h had a larger specific surface area and pore volume than other samples, which was mainly caused by its hollow morphology. The higher specific surface area can reduce the diffusion resistance and provide more active sites to promote the photocatalytic reaction, thereby the adsorption capacity of the photocatalyst for pollutants is improved [35].

The generation of photoluminescence is mainly caused by the recombination of photogenerated carriers, and the photoluminescence spectrum is used to reflect the separation efficiency of electrons and holes of the catalysts [36,37]. In general, the generation of fluorescence is caused by electron–hole recombination. The higher the fluorescence intensity, the more serious the electron–hole recombination of the semiconductor and the weaker the photocatalytic performance. As shown in Figure 7b, with the increase in calcination temperature and time, the fluorescence intensity of the sample gradually weakened. At room temperature, 600 °C/7 h had the lowest fluorescence intensity. It was speculated that this unique hollow structure is beneficial to the transport and separation of photon carriers.

**Figure 8:**
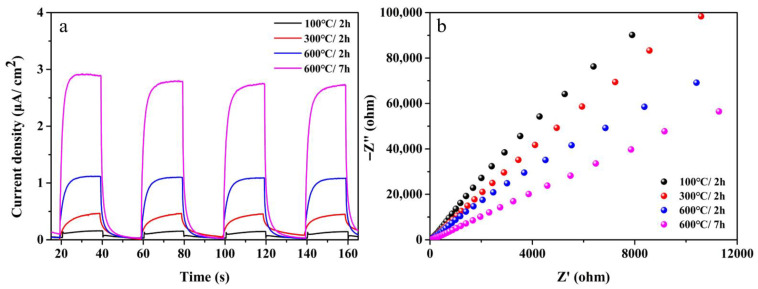
(**a**) Transient photocurrent response tests of all samples and (**b**) EIS Nyquist plots of all samples. Note: 100 °C/2 h, the precursor calcined at 100 °C for 2 h; 300 °C/2 h, the precursor calcined at 300 °C for 2 h; 600 °C/2 h, the precursor calcined 600 °C for 2 h; 600 °C/7 h, the precursor calcined 600 °C for 7 h.

Transient photocurrent response and EIS Nyquist plots were provided to characterize the electrochemical performance of the as-prepared samples under different calcination conditions. It can be seen in Figure 8a that the prepared TiO_2_ with the hexagonal frame structure had a high transient photocurrent intensity. The sizes of the arc radii of the EIS Nyquist plots (Figure 8b) were 600 °C/7 h < 600 °C/2 h < 300 °C/2 h < 100 °C/2 h. The smaller the arc radius, the larger the charge transfer resistance and the charge transfer is also limited [38]. Therefore, it could be concluded from the two electrochemical test results that when the calcination condition was 600 °C/7 h, the hexagonal frame structure TiO_2_ had excellent charge separation performance, thereby improving the photocatalytic performance. This also corresponded to the PL test as mentioned above.

**Figure 9:**
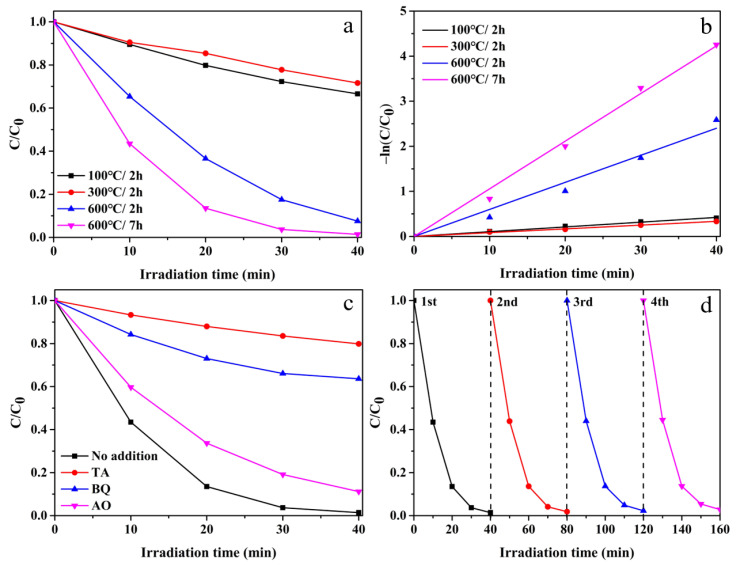
(**a**) Photocatalytic degradation of RhB using four samples under UV-vis light, (**b**) linear fit between −ln (C/C_0_) and time among four samples, (**c**) trapping experiments of active species and (**d**) cyclic degradation curve of TiO_2_ with annealing 600 °C for 7 h. Note: 100 °C/2 h, the precursor calcined at 100 °C for 2 h; 300 °C/2 h, the precursor calcined at 300 °C for 2 h; 600 °C/2 h, the precursor calcined 600 °C for 2 h; 600 °C/7 h, the precursor calcined 600 °C for 7 h.

The photocatalytic activity of the prepared samples was evaluated using photocatalytic degradation of RhB under UV-vis light (Figure 9a). After 40 min of light irradiation, the degradation efficiency of 600 °C/7 h reached 99%, while the degradation efficiencies of 100 °C/2 h, 300 °C/2 h and 600 °C/2 h were 33%, 28% and 92%, respectively. The Langmuir–Hinshelwood model is often used to illustrate the kinetics of heterogeneous photocatalytic processes. The specific equation is as follows:

C = C_0_e^−kt^ (4)

−ln(C/C_0_) = kt (5)

where C_0_ (mg/L) is represented as the concentration of RhB aqueous solution at irradiation time t = 0 min. C (mg/L) is represented as the concentration of the RhB aqueous solution at irradiation time t (min). k (min^−1^) is represented as the first-order rate constant [39]. The rate constant k was calculated through a plot of linear fit between −ln(C/C_0_) against irradiation time (Figure 9b). The linear graph showed that the degradation process of RhB by TiO_2_ was in good agreement with the Langmuir–Hinshelwood first-order kinetic equation. It can be seen that the photocatalytic degradation rate constant of 600 °C/7 h was 0.106 min^−1^, which was higher than 100 °C/2 h (0.011 min^−1^), 300 °C/2 h (0.008 min^−1^) and 600 °C/2 h (0.06 min^−1^). The hollow structure enabled it to have a larger specific surface area compared with other samples. There were more reactive sites on its surface [40]. The rate constant can be improved by increasing the number of active sites during photocatalytic degradation [41,42]. It also promoted the adsorption of RhB dye on hollow hexagonal frame TiO_2_. In addition, the excellent photocatalytic activity was also related to high crystallinity. Compared with other morphologies of TiO_2_ prepared in previous studies, the TiO_2_ with hollow hexagonal morphology had more excellent photocatalytic degradation activity (Table S1). The radical trapping experiment of catalysts was carried out (Figure 9c). Compared with no scavenger added, it was clearly found that the photocatalytic degradation rate of RhB was decreased after adding BQ or TA, proving that ·OH and ·O_2_^−^ played a major role in the photocatalytic activity. The recyclability and stability evaluation of 600 °C/7 h is shown in Figure 9d, and it can be seen that the catalyst still maintained a good degradation efficiency after four cycles, indicating that the hollow hexagonal frame TiO_2_ has excellent potential for application in the field of wastewater treatment.

In order to study the effects of different dosages of catalyst on the degradation of the RhB solution, different amounts of 600 °C/7 h sample (0.05 g, 0.10 g, 0.15 g and 0.20 g) were added to 100 mL of RhB under basic conditions. It can be seen from Figure S1 that when the amount of catalyst increased from 0.10 g to 0.20 g, the degradation rate decreased from 98.6% to 86.3% and the rate constant also changed from 0.106 min^−1^ to 0.049 min^−1^. When the high dosage of catalyst was added, part of the catalyst surface could not perform photon absorption and dye adsorption due to the shielding effect [43]. The worst degradation effect was obtained when 0.05 g catalyst was added, mainly because the number of active sites was insufficient, and it was difficult to adsorb more dye. Therefore, it was considered to be the optimal condition when the dosage of the catalyst was 0.10 g.

**Figure 10:**
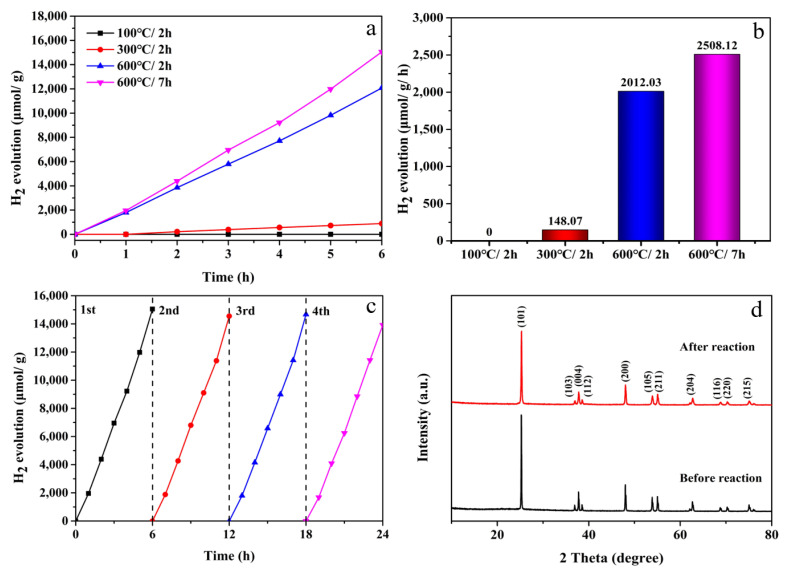
(**a**) Photocatalytic H_2_ yield, (**b**) H_2_ evolution rate, (**c**) cycling test of photocatalytic H_2_ generation of TiO_2_ with annealing at 600 °C for 7 h and (**d**) XRD patterns of original and recycling samples of recycling samples. Note: 100 °C/2 h, the precursor calcined at 100 °C for 2 h; 300 °C/2 h, the precursor calcined at 300 °C for 2 h; 600 °C/2 h, the precursor calcined 600 °C for 2 h; 600 °C/7 h, the precursor calcined 600 °C for 7 h.

To further verify the excellent photocatalytic performance of the as-prepared hexagonal frame TiO_2_, a series of experiments on photocatalytic hydrogen evolution were carried out. With methanol as the sacrificial agent and chloroplatinic acid added, [PtCl_6_]^2^^−^ in the solution was reduced to elemental Pt by photoelectrons generated by photoexcitation of TiO_2_ conduction band under UV-vis light irradiation [44]. With Pt-decorated TiO_2_ as a co-catalyst for hydrogen evolution, the rate of hydrogen evolution could be improved effectively. As shown in Figure 10a, the hydrogen yield of 100 °C/1 h within 6 h was 0. With the increase in calcination temperature and calcination time, the photocatalytic activity of the sample gradually increased. It can be seen from Figure 10b that 600 °C/7 h had the highest hydrogen yield at 2508.12 μmol g^−1^ h^−1^, which was significantly higher than the hydrogen yields of 100 °C/2 h (0 μmol g^−1^ h^−1^), 300 °C/2 h (148.07 μmol g^−1^ h^−1^) and 600 °C/2 h (2012.03 μmol g^−1^ h^−1^). This was also due to the high crystallinity and large specific surface area of 600 °C/7 h. All these reasons improved the photocatalytic hydrogen evolution performance. Under the same conditions, 600 °C/7 h was tested for four cycles of hydrogen production, and the cycle was repeated every 6 h. As shown in Figure 10c, the hydrogen production capacity of the sample changed little after four cycles. This showed that 600 °C/7 h had good stability. The samples after the hydrogen evolution reaction were analyzed using XRD (Figure 10d) and the diffraction peaks basically did not change, which further demonstrated the stability of the sample.

**Figure 11:**
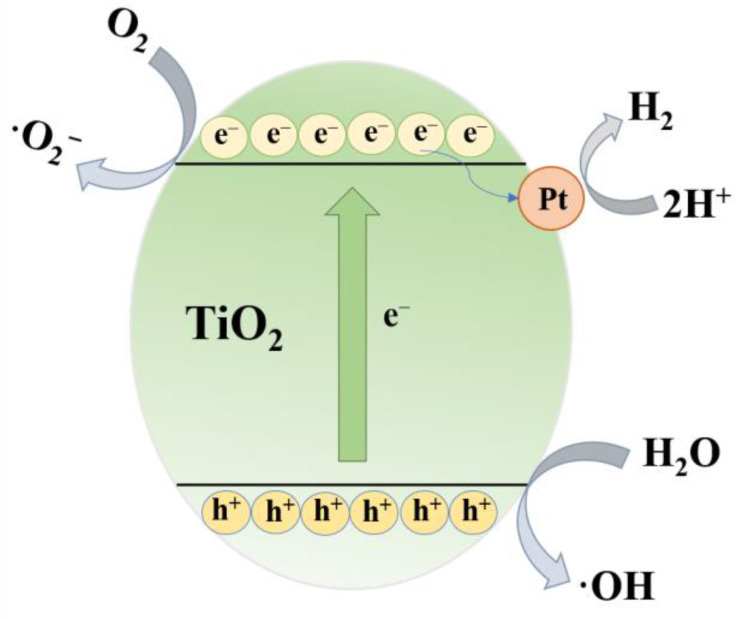
Photocatalytic mechanism of hexagonal frame TiO_2_ (e^−^ represents an electron; h^+^ represents a hole).

The photocatalytic mechanism is presented in Figure 11. It can be seen that when TiO_2_ is irradiated by light, electrons (e^−^) in the valence band (VB) are excited to the conduction band (CB), thereby generating photoinduced holes (h^+^) in the valence band. The generated carriers migrate to the catalyst surface under the action of the electric field. These carriers can react with oxygen and water on the surface of TiO_2_ to generate ·O_2_^−^ and ·OH, thus realizing the degradation of RhB dyes. In the photocatalytic hydrogen evolution reaction, the cocatalyst Pt can capture the e^−^ in the CB. The h^+^ reacts with water in the solution to form H^+^, which is reduced to H_2_ by e^−^. On the one hand, the hydrogen evolution cocatalyst Pt captures e^−^ and delays the recombination with h^+^. On the other hand, methanol as a sacrificial agent can react with h^+^ and inhibit the recombination of photogenerated carriers [45]. Both of these reasons can improve the photocatalytic H_2_ evolution performance of the catalyst.

## 4. Conclusions

In conclusion, a template-free method was used to synthesize hollow hexagonal frame TiO_2_. The first step was to synthesize the precursor (HTiOF_3_) under the hydrothermal method and then calcine it at a certain temperature and time to obtain TiO_2_. By changing the type of solvent, the influencing factors of the precursor morphology were explored, and it was further demonstrated that the spherical structure composed of hexagonal prisms was obtained with certain conditions. In addition, the transformation process from the precursor to TiO_2_ was also studied by changing the calcination temperature and time. The highest photocatalytic activity was found in the hollow hexagonal frame TiO_2_, which was due to the larger specific surface area of the hollow structure. Through the photocurrent, EIS and PL measurements analysis, it was confirmed that the hexagonal frame TiO_2_ had a lower recombination rate of photogenerated electrons and holes. The excellent photocatalytic performance of TiO_2_ could be more intuitively shown by photocatalytic degradation of RhB solution and photocatalytic hydrogen evolution experiments.

## Figures and Tables

**Table 1:** The composition of the solvent under different reaction conditions.

Sample Number	TBT (mol)	Titanium Tetraisopropanolate (mol)	HAc (mL)	Isopropanol (mL)	HF (mL)
S1	0.1	0	28	21	1.6
S2	0.1	0	0	21	1.6
S3	0.1	0	28	0	1.6
S4	0.1	0	28	21	0
S5	0	0.01	28	21	1.6

**Table 2:** BET surface area and pore volume of all samples.

Sample	BET Surface Area (m^2^/g)	Pore Volume (cm^3^/g)
100 °C/2 h ^1^	23.2	0.152
300 °C/2 h ^2^	41.6	0.221
600 °C/2 h ^3^	48.7	0.253
600 °C/7 h ^4^	51.9	0.571

^1^ The precursor calcined at 100 °C for 2 h. ^2^ The precursor calcined at 300 °C for 2 h. ^3^ The precursor calcined at 600 °C for 2 h. ^4^ The precursor calcined at 600 °C for 7 h.

## Data Availability

Data supporting this study are available within the article.

## Supplementary Materials

The following are available online at https://www.mdpi.com/article/10.3390/nano12091409/s1, Figure S1: (a) Photocatalytic degradation of RhB under UV light with different amounts of catalyst; (b) Linear fit between −ln (C/C_0_) and time of different amounts of catalyst, Table S1: Comparison of photocatalytic degradation of organic dyes catalyzed by TiO_2_ catalysts. References [46,47,48,49,50,51] are cited in the supplementary materials.
