# Supplementary material for: Preparation and Photocatalytic Properties of Anatase TiO2 with Hollow Hexagonal Frame Structure
Source: Nanomaterials (Basel). 2022 Apr 20;12(9):1409. doi: 10.3390/nano12091409 (PMC9104110; doi:10.3390/nano12091409)
Supplement: Supplementary file 1 [file nanomaterials-12-01409-s001.zip › nanomaterials-1682534-supplementary.pdf]

Supplementary Materials

Preparation and Photocatalytic Properties of Anatase TiO_2 with Hollow Hexagonal Frame Structure

Mengyuan Teng, Haixia Liu *, Bensheng Lin, Xiangzhu Zhou and Wei Zhou *

Characterization data

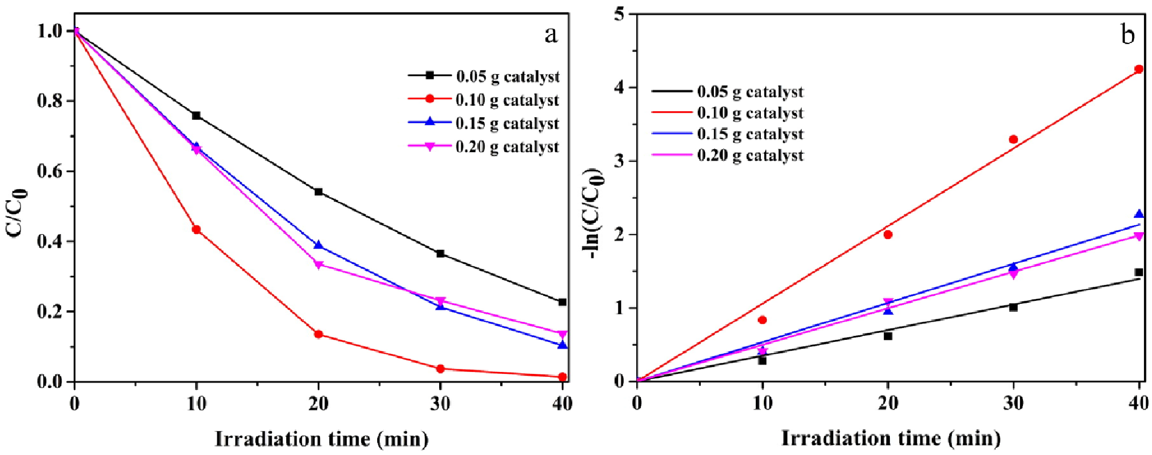

Figure S1. (a) Photocatalytic degradation of RhB under UV light with different amounts of catalyst; (b) Linear fit between −ln (C/C_0) and time of different amounts of catalyst.

Table. S1 Comparison of photocatalytic degradation of organic dyes catalyzed by TiO_2 catalysts

No.	Catalyst	Dyes	Reaction conditions	Degradation Rate.	Ref.
1	TiO_2 microspheres	Methylene Blue (MB)	*c*(MB) = 5.0 mg/L, *c*(Cat.) = 1.0 g/L, 60 min, UV light	99.0 %	[1]
2	TiO_2 nanotubes	Rhodamine B (RhB)	*c*(RhB) = 10.0 mg/L, *c*(Cat.) = 0.2 g/L, 100 min, UV-Vis. light	95.8 %	[2]
3	tube-in-tube TiO_2 nanotubes	MB	*c*(MB) = 10^{-5} mol/L, *c*(Cat.) = 0.3 g/L, 180 min, UV light	77.0 %	[3]
4	TiO_2 hollow spheres	Methyl Orange (MO)	*c*(MO) = 10^{-5} mol/L, *c*(Cat.) = 0.4 g/L, 140 min, UV light	89.7 %	[4]
5	3D hierarchical flower-like TiO_2	RhB	*c*(RhB) = 10.0 mg/L, *c*(Cat.) = 1.0 g/L, 60 min, UV light	90.0 %	[5]

	nanospheres				
6	ultrathin TiO_2 nanosheets	RhB	*c*(RhB) = 10.0 mg/L, *c*(Cat.) = 1.0 g/L, 60 min, UV light	99.0 %	[6]
7	hollow hexagonal frame TiO_2	RhB	*c*(RhB) = 20 mg/L, *c*(Cat.) = 1 g/L, 40 min, UV-Vis light	99.0 %	Our work

1. Ruqaishy, M.A.; Marzouqi, F.A.; Qi, K.Z.; Liu, S.Y.; Karthikeyan, S.; Kim, Y.; Al-Kindy, S.M.Z.; Kuvarega, A.T.; Selvaraj, R. Template-free preparation of TiO_2 microspheres for the photocatalytic degradation of organic dyes. Korean J. Chem. Eng. 2018, 35, 2283-2289.
2. Wang, T.; Gao, Y.; Tang, T.; Bian, H.Q.; Zhang, Z.M.; Xu, J.H.; Xiao, H.; Chu, X. Preparation of ordered TiO_2 nanofibers/nanotubes by magnetic field assisted electrospinning and the study of their photocatalytic properties. Ceram. Int. 2019, 45, 14404-14410.
3. Xu, X.J.; Fang, X.S.; Zhai, T.Y.; Zeng, H.B.; Liu, B.D.; Hu, X.Y.; Bando, Y.; Golberg, D. Tube-in-Tube TiO_2 nanotubes with porous walls: Fabrication, formation mechanism, and photocatalytic properties. Small 2011, 7, 445-449.
4. Kanjana, N.; Maiaugree, W.; Poolcharuansin, P.; Laokul, P. Size controllable synthesis and photocatalytic performance of mesoporous TiO_2 hollow spheres. J. Mater. Sci. Technol. 2020, 48, 105-113.
5. Du, Y.B.; Xu, X.Y.; Lin, L.; Ge, M.Y.; He, D.N. 3D hierarchical flower-like rutile TiO_2 nanospheres-based versatile photocatalyst. J. Mater. Sci. 2018, 53, 385-395.
6. Chen, K.M.; Jiang, Z.; Qin, J.L.; Jiang, Y.; Li, R.; Tang, H.; Yang, X.F. Synthesis and improved photocatalytic activity of ultrathin TiO_2 nanosheets with nearly 100% exposed (001) facets. Ceram. Int. 2014, 40, 16817-16823.
